# Effects of Maternal Obstructive Sleep Apnoea on Fetal Growth: A Prospective Cohort Study

**DOI:** 10.1371/journal.pone.0068057

**Published:** 2013-07-24

**Authors:** Alison M. Fung, Danielle L. Wilson, Martha Lappas, Mark Howard, Maree Barnes, Fergal O'Donoghue, Stephen Tong, Helen Esdale, Gabrielle Fleming, Susan P. Walker

**Affiliations:** 1 Department of Perinatal Medicine, Mercy Hospital for Women, Melbourne, Australia; 2 University of Melbourne, Department of Obstetrics and Gynaecology, Mercy Hospital for Women, Melbourne, Australia; 3 Institute for Breathing and Sleep, Austin Health, Melbourne, Australia; 4 Department of Medicine, University of Melbourne, Melbourne, Australia; Université de Montréal, Canada

## Abstract

**Objective:**

The objective of this study is to determine whether obstructive sleep apnea (OSA) is associated with reduced fetal growth, and whether nocturnal oxygen desaturation precipitates acute fetal heart rate changes.

**Study Design:**

We performed a prospective observational study, screening 371 women in the second trimester for OSA symptoms. 41 subsequently underwent overnight sleep studies to diagnose OSA. Third trimester fetal growth was assessed using ultrasound. Fetal heart rate monitoring accompanied the sleep study. Cord blood was taken at delivery, to measure key regulators of fetal growth.

**Results:**

Of 371 women screened, 108 (29%) were high risk for OSA. 26 high risk and 15 low risk women completed the longitudinal study; 14 had confirmed OSA (cases), and 27 were controls. The median (interquartile range) respiratory disturbance index (number of apnoeas, hypopnoeas or respiratory related arousals/hour of sleep) was 7.9 (6.1–13.8) for cases and 2.2 (1.3–3.5) for controls (p<0.001). Impaired fetal growth was observed in 43% (6/14) of cases, vs 11% (3/27) of controls (RR 2.67; 1.25–5.7; p = 0.04). Using logistic regression, only OSA (OR 6; 1.2–29.7, p = 0.03) and body mass index (OR 2.52; 1.09–5.80, p = 0.03) were significantly associated with impaired fetal growth. After adjusting for body mass index on multivariate analysis, the association between OSA and impaired fetal growth was not appreciably altered (OR 5.3; 0.93–30.34, p = 0.06), although just failed to achieve statistical significance. Prolonged fetal heart rate decelerations accompanied nocturnal oxygen desaturation in one fetus, subsequently found to be severely growth restricted. Fetal growth regulators showed changes in the expected direction- with IGF-1 lower, and IGFBP-1 and IGFBP-2 higher- in the cord blood of infants of cases vs controls, although were not significantly different.

**Conclusion:**

OSA may be associated with reduced fetal growth in late pregnancy. Further evaluation is warranted to establish whether OSA may be an important contributor to adverse perinatal outcome, including stillbirth.

## Introduction

Detection of intrauterine growth restriction (IUGR) remains a leading priority in obstetric care, where the combined efforts of aggressive *in utero* surveillance and timely delivery are necessary to prevent stillbirth. After appropriate adjusting for constitutional variance, more than one half of unexplained stillbirths have been confirmed to be associated with fetal growth restriction [1], [2]. Impairment of fetal growth may have maternal or placental contributors. Maternal contributors include hypertension [3] and conditions associated with hypoxia, such as cardio-respiratory disease, or living at high altitude [4]. Placental factors include impaired vascular perfusion [5] or inflammatory activation [6] that may contribute to impaired oxygen and nutrient exchange.

A condition that shares the pathologies of hypoxia, sympathetic activation and systemic inflammation is obstructive sleep apnoea (OSA). OSA occurs when the upper airway collapses during sleep, resulting in cessation of breathing, and is accompanied by episodic hypoxia and hypercapnia. Furthermore, OSA activates the sympathetic nervous system [7] and inflammatory pathways [8], [9]. It is therefore plausible that, through these mechanisms, OSA may be an unsuspected contributor to fetal growth restriction and stillbirth. If this association was confirmed, it would be potentially important, given there is safe and effective treatment in the form of Continuous Positive Airway Pressure (CPAP). Conceivably, CPAP could be a novel treatment to decrease the burden of IUGR.

Therefore, in this study, we examined whether the presence of OSA, confirmed on sleep study, was associated with impaired fetal growth in late pregnancy, and alterations in fetal growth hormones (the insulin like growth factor axis). Secondly, we examined whether periods of maternal oxygen desaturation overnight were accompanied by acute fetal heart rate changes.

## Materials and Methods

This was a prospective longitudinal study conducted between May 2009 and November 2011 at the Mercy Hospital for Women, a tertiary obstetric centre in Melbourne, Australia, responsible for approximately 5,800 deliveries/year, and at the Institute for Breathing and Sleep, Austin Health, Melbourne. The study was approved by the Institutional Review Boards at the Mercy Hospital for Women and Austin Health. Written informed consent was obtained for all participants.

Convenience sampling was performed of women in the second trimester of pregnancy at the time of their routine antenatal visit. They were asked to complete a preliminary screening questionnaire for symptoms of Sleep Disordered Breathing (SDB). The screening questionnaires used were the Berlin Questionnaire [10] and Multivariable Apnoea Risk Index [11] (MAP Index), both of which are validated tools to screen for symptoms of sleep disordered breathing in non-pregnant populations. The questionnaires address symptoms such as snoring, gasping, witnessed apnoeas and excessive daytime somnolence. These are scored depending on the frequency of self-reported symptoms. While sensitive [12], such screening questionnaires lack specificity for the diagnosis of OSA [10], and studies to date suggest a further reduction in performance in pregnant women [13], [14].

Accordingly, we sought to confirm the diagnosis by performing formal sleep studies in a selected subset of respondents. 51 women between 24 and 32 weeks gestation (confirmed by early pregnancy ultrasound) with singleton uncomplicated pregnancies were recruited to the longitudinal study. These women comprised a convenience sample from the larger cohort, selected to represent women with both ‘low-risk’ and ‘high-risk’ questionnaire scores. A ‘high-risk’ score required scoring high in at least 2 out of 3 categories of the Berlin questionnaire (snoring and witnessed apnoeas, daytime or driving sleepiness, history of high blood pressure or early pregnancy BMI more than 30 kg/m^2^). Those who denied having persistent symptoms (>3–4 times/week), or who qualified for only one symptom category, were placed in the ‘low risk’ group. Given the sleep study was being performed at term, women were only eligible for the longitudinal study if their pregnancy was otherwise considered to be low risk, without other co-morbidities, such as multiple pregnancy, fetal abnormality, pre-existing hypertension or diabetes

All women in the longitudinal study underwent an ultrasound for fetal growth at 32 weeks' gestation, including amniotic fluid volume and umbilical artery Doppler assessments. These ultrasounds were performed by a single investigator (AF) who was blinded to the results of the screening questionnaire. The ultrasound estimate of fetal weight was customised for maternal height, pre-pregnancy (or if unknown, early pregnancy) weight, ethnicity, parity and fetal gender using the Australian dataset of the GROW software (www.gestation.net) [15]. Customisation of estimated fetal weight (in utero) and birthweight (following delivery) is superior to population based weight centiles in studies evaluating fetal growth as it generates an in utero growth standard, which is then individualised for each fetus, to adjust for relevant maternal characteristics which may affect fetal growth. Thirty-two weeks was selected for the growth ultrasound, to enable sufficient time between third trimester growth assessment and delivery for any significant change in customised centile across the third trimester to be detected, and slowing of fetal growth trajectory identified.

Overnight polysomnography (PSG, a ‘sleep study’) was performed in either the sleep laboratory or the woman's home, depending on her preference. The PSG was used to establish the presence or absence of OSA, using the respiratory disturbance index (RDI). The RDI is the number of apnoeas (cessation of airflow ≥10 seconds), hypopnoeas (reduction in airflow ≥10 seconds associated with an oxygen desaturation of ≥3% or an arousal) and respiratory event-related arousals (RERAs; a sequence of breaths lasting ≥10 seconds associated with flattening of the nasal pressure waveform leading to an arousal from sleep) recorded per hour of sleep [16]. OSA was defined as an RDI of 5 or more [17], [18]. While oxygen desaturation is not necessary for the diagnosis of OSA using this classification, the number of oxygen desaturations of ≥3% and ≥4% per hour of sleep was also calculated. PSG was performed using the Somté PSG (Compumedics, Abbotsford, Australia) portable sleep-monitoring device. Signals measured included; electroencephalogram (C4-A1), left and right electro-oculogram, nasal airflow (using a nasal cannula), arterial oxygen saturation, thoracic and abdominal respiratory effort, body position, and heart rate. PSG recordings were sleep staged and respiratory scored by a single experienced sleep technologist (DW). The sleep study was scored in accordance with current American Academy of Sleep Medicine criteria [18], with the alternative definition used for scoring hypopnoeas. The RDI was calculated, and cases were defined as women with an overall RDI ≥5/hr, and controls were defined as women with an RDI<5/hr.

Time-synchronised continuous electronic fetal heart rate monitoring (CEFM) was performed using the Monica AN24 fetal heart rate monitor (Monica Healthcare Ltd) during the overnight sleep study to enable correlation of any fetal heart rate abnormality with objectively confirmed respiratory events. All fetal heart rate traces were reviewed the following morning by a single observer (AF) blinded to the questionnaire or PSG results. *A priori* criteria for an ‘abnormal fetal heart rate monitoring event’ were as follows: (i) *prolonged bradycardia*; fetal heart rate >15 beats/min below baseline for ≥90 seconds and lasting <5 minutes, (ii) *recurrent severe variable decelerations*; a fall of >60 beats per minute from previous baseline heart rate and of >60 seconds duration *and* at least 2 per 2 hours, and (iii) *repeated unprovoked (in the absence of contractions) or late decelerations accompanied by tachycardia or loss of variability*. Abnormal fetal heart rate monitoring events were reported to the treating doctor for determination of further management.

The sleep studies were all performed at approximately 37 weeks' gestation. This was to avoid the potential complication of provoking iatrogenic prematurity if abnormal fetal heart rate monitoring was detected at a preterm gestation. This was considered necessary by the Research Ethics Committee in view of a (then, recently) published paper reporting abnormal fetal heart monitoring during overnight sleep studies in 3 out of 4 fetuses of women with OSA [19]. Accordingly, case assignment was at completion of the pregnancy, which further ensured that those performing ultrasound assessment of fetal growth were blinded to case or control status.

At the time of delivery, birthweight (BW) was customised as previously described, using the GROW software (www.gestation.net) [15], so that fetal size at both assessments was adjusted for maternal BMI and other characteristics. *Fetal growth restriction* (FGR) was defined as a customised birthweight <10^th^ centile for gestational age. Evidence of s*lowing third trimester growth* was defined as a fall in customised centile of greater than a third (33% decrease) between the 32-week ultrasound and birth. *Impaired fetal growth* was defined as *either* FGR or slowing third trimester growth. Cord blood was also collected at delivery and was analysed for the fetal growth regulators IGF-1, IGF-2, IGFBP-1 and IGFBP-2. IGF-1 determination was performed using MILLIPLEX MAP human IGF-1 single plex kit, (Millipore, Billerica, MA, USA). The assay utilised 25 µl of plasma at 1/8 dilution. The limit of detection was 137 pg/ml. Assays were read using the Bio-Plex workstation (Bio-Rad Laboratories, Hercules, CA. For IGF-2, we used an IGF-2 ELISA from Demeditec Diagnostics (Kiel-Wellsee, Germany), with a limit of detection of 0.2 ng/ml. All samples assayed at 1/50 dilution. Plates were read at 450 nm with wavelength correction at 620 nm using a Bio-Rad microplate reader (xMarkT Microplate Absorbance Reader, Bio-Rad Laboratories, Hercules, CA, USA). IGFBP determination was performed using MILLIPLEX MAP (multi-analyte panel) human IGFBP panel kits (Millipore, Billerica, MA, USA). The assay utilised 25 µl of plasma at 1/25 dilution. The limit of detection varied for each IGFBP and ranged from 0.03–1.4 ng/ml.

The sample size was based upon the original observations of Sahin et al [19], that 75% of patients with OSA confirmed on polysomnography had significant fetal heart decelerations overnight. Assuming the incidence of decelerations was indeed 75% in cases, and ≤20% in controls, we required 12 patients with OSA to be confirmed on PSG. We calculated that this would likely require 25 ‘high risk’ patients on questionnaire screening to be recruited to the longitudinal study. Means with standard deviations (or medians with interquartile range) were used for descriptive statistics for continuous variables, and proportions for categorical variables. The incidence of impaired fetal growth was compared between cases and controls using the Fisher's exact test. Logistic regression was then used to examine the relationship between OSA, BMI, hypertensive disorders of pregnancy (gestational hypertension or pre-eclampsia) and gestational diabetes on fetal growth impairment. Stepwise multivariate logistic regression was used to adjust for any residual confounding, with all variables with a p value of <0.1 on univariate analysis included in the multivariate model. The median RDI between pregnancies complicated by impaired fetal growth and normal fetal growth was compared using the Mann Whitney U test. Mean cord blood IGF-1, IGF-2 and their respective binding proteins were compared between cases and controls using the Student t-test. Significance was taken at p<0.05. All statistical analyses were performed with SPSS 17.0 (SPSS Inc., Chicago, Illinois).

## Results

371 women completed screening questionnaires for symptoms of OSA at a mean gestational age of 21.4 (SD 2.4) weeks. Their mean age was 31.2 years, pre-pregnancy body mass index (BMI) 26.1 (SD 6.4), and 153 (41%) of women were nulliparous. Of the 371 women, 108 (29%) qualified as being high risk for OSA, and 263 (71%) were low risk.

**Figure 1:**
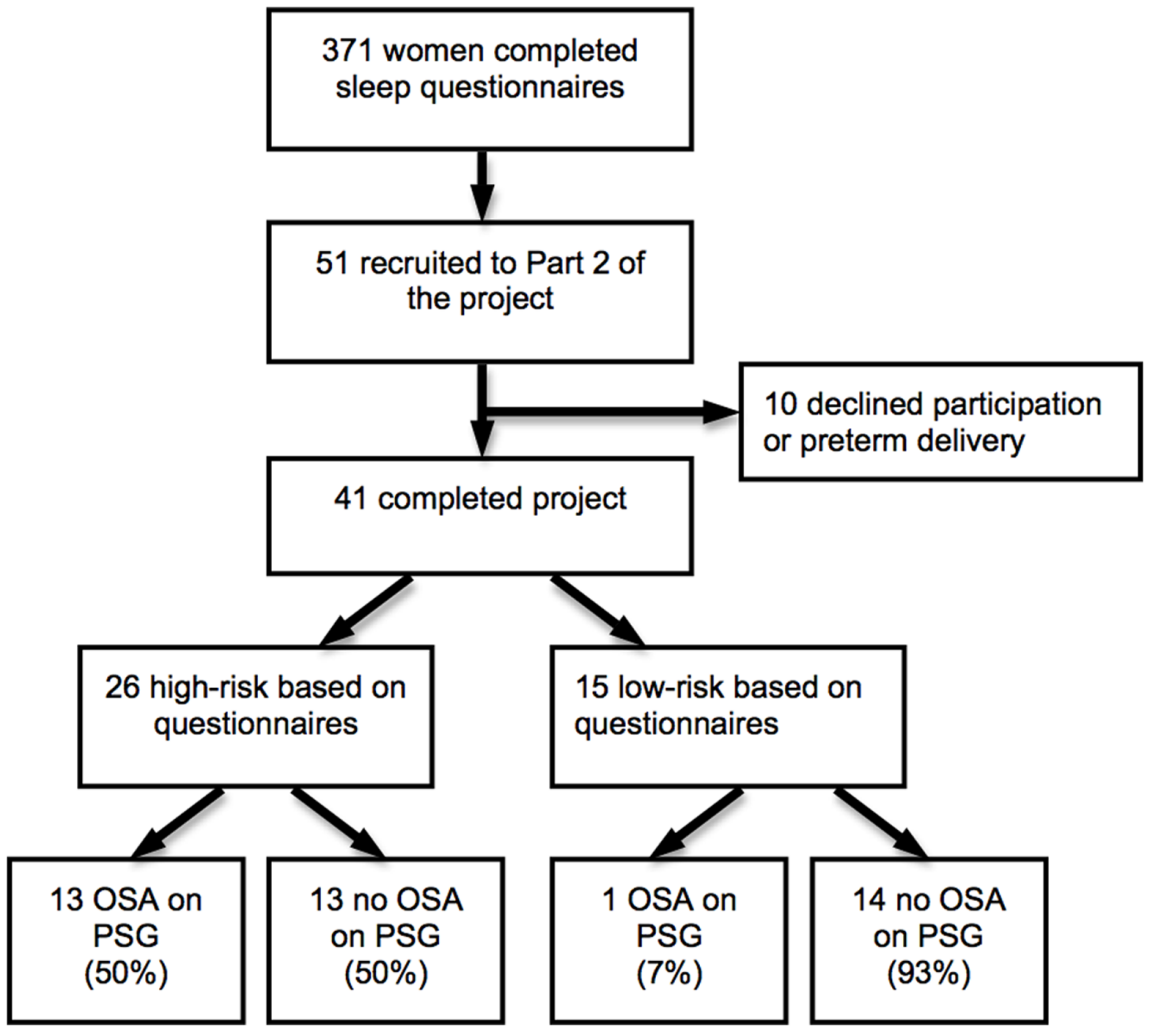
Participant flow diagram.

Of the 51 women who agreed to participate in the longitudinal study, four were subsequently excluded due to preterm delivery, prior to their polysomnography (2 were high risk on questionnaire and 2 were low risk), and 6 women withdrew from the study. Accordingly, 41 women (26 who were high risk on screening, and 15 who were low risk on screening) completed all assessments of fetal growth and had a diagnostic sleep study performed at 37 weeks. Of these, 14 women met the study-defined criteria for OSA and 27 did not have OSA (median RDI 7.9 vs 2.2, p<0.001). Among women who scored ‘high risk’ on the Berlin questionnaire at the time of recruitment, the sensitivity and specificity for detection of OSA (RDI>5) was 93% and 48%, respectively. Figure 1 demonstrates the participant flow diagram.

Women with OSA had a higher respiratory disturbance index (RDI), apnoea hypopnea index (AHI), and had more oxygen desaturations of ≥3%, and ≥4% than controls, as well as having greater drops in oxygen saturation, lower minimum oxygen saturations and a greater proportion of total sleeping time (TST) with oxygen saturation below 90%. The respiratory data on cases and controls is summarised in Table 1.

**Table 1:** Summary of Respiratory Data: cases versus controls.

	Cases (n = 14)	Controls (n = 27)	P value
Respiratory Disturbance Index	7.9 (6.1–13.8)	2.2 (1.3–3.5)	<.001
Apnoea Hypopnoea Index	6.2 (4.9–11.7)	1.4 (0.6–2.6)	<.001
Oxygen Desaturation Index ≥3%	3.4 (1.5–9.4)	0.4 (0.1–1.0)	<.001
Oxygen Desaturation Index ≥4%	1.9 (0.7–5.1)	0.1 (0.0–0.3)	<.001
Number of desaturations ≥3%	16.5 (9.3–38.5)	3.0 (1.0–5.0)	<.001
Number of desaturations ≥4%	9.5 (4.0–23.5)	1.0 (0.0–2.0)	<.001
Largest oxygen desaturation (%)	6 (5.0–8.3)	4 (3.0–5.0)	.001
Minimum O2 (%)	90 (88–90.3)	91 (90–93)	.044
%Total Sleep Time below 95%	34.7 (12.0–58.2)	17.2 (4.4–56.5)	.25
%Total Sleep Time below 90%	0.1 (0–0.1)	0 (0–0)	.003

Median (Interquartile Range).

The clinical and pregnancy characteristics of these women are summarised in Table 2. Nine pregnancies were complicated by impaired fetal growth; 5 fetuses had a birthweight <10^th^ centile at delivery, and 4 fetuses exhibited a significant slowing of fetal growth trajectory in the third trimester. At 32 weeks, all fetuses were appropriately grown with normal umbilical artery Doppler blood flow studies and biophysical profile.

**Table 2:** Clinical characteristics of patients with OSA (cases) and controls.

Clinical characteristics	Cases n = 14	Controlsn = 27	*P* value
Age (years)	36.0 (4.4)	33.4 (4.8)	0.10
Parity	0.8 (0.6)	1.0 (0.8)	0.37
BMI (kg/m^2^) pre-pregnancy	35.1 (5.4)	31.0 (8.9)	0.13
Smoker	2 (14%)	3 (12%)	1.0
**Pregnancy complications**			
Gestational hypertension	2 (14%)	6 (22%)	0.69
Preeclampsia	1 (7%)	1 (4%)	1.0
Gestational diabetes	4 (29%)	3 (12%)	0.21
**Perinatal Outcome**			
Gestation at delivery (weeks)	38.7(1.0)	39.4 (1.3)	0.06
Birthweight (grams)	3378(472)	3567(501)	0.25
Birthweight centile	47 (29)	54 (30)	0.49
Impaired fetal growth (Birthweight <10^th^ centile **or** fall in customised centile >33% between 32 weeks and term)	6 (43%)	3 (11%)	0.04
*Birthweight <10^th^ centile*	2 (14%)	3 (11%)	1
*Fall in customised centile >33% between 32 weeks and term*	4 (29%)	0 (0%)	<0.01
Apgar ≤7 at 5 min	0	1 (4%)	1.0
Admission SCN/NICU	0	1 (3.7%)	1.0

Data presented as mean (SD) or number (%).

Among the women with OSA (RDI≥5), 43% (6/14) demonstrated impaired fetal growth across the third trimester, compared with 11% (3/27) of controls (RR 2.67; 1.25–5.7; p = 0.04). Of these 6 women, 2 delivered an infant less than the 10^th^ centile (IUGR), and 4 had a fetus that demonstrated slowing of fetal growth between 32 weeks and birth. Of the 27 control women, 3/27 (11%) delivered an infant less than the 10^th^ centile, and 0/27 (0%) demonstrated slowing of fetal growth between 32 weeks and birth. On logistic regression, univariate analysis revealed the only significant predictors of impaired fetal growth were OSA (OR 6; 1.2–29.7; p = 0.03) and BMI (OR 2.52; 1.09–5.80; p = 0.03). There was no significant association observed with impaired fetal growth and either hypertensive disorders of pregnancy or gestational diabetes in this small, low risk cohort (Table 3). Multivariate logistic regression confirmed minimal change to the association between RDI>5 and impaired fetal growth (OR 5.3; 0.93–30.34; p = 0.06) after adjusting for any potential residual confounding by BMI (Table 4). The median RDI among all women with fetal growth impairment in the cohort (n = 9) was significantly higher than those demonstrating normal growth (n = 32); RDI 6.6 vs 3.5, p = 0.04.

**Table 3:** Factors associated with Impaired Fetal Growth.

Variable	Units or Category (*Range/SD*)	OR (*95% CI*)	*p* value
RDI greater than 5	Yes or No	6.00 (1.2–29.7)	.028
Pre-pregnancy BMI*	kg/m^2^ (20.6–56.8/8.0)	2.52 (1.09–5.80)	.03
GH or Pre-eclampsia	Yes or No	1.79 (0.35–9.02)	.48
GDM	Yes or No	3.38 (0.59–19.21)	.17

*Note.* OR = odds ratio; BMI = body mass index; GH = gestational hypertension; GDM = gestational diabetes mellitus.

* OR for continuous variables indicate the change in odds for an increase of one standard deviation.

**Table 4:** Factors associated with Impaired Fetal Growth on Stepwise Logistic Regression Model.

Variable	Coefficient	OR (95% CI)	*p* value
RDI greater than 5	1.67	5.30 (0.93–30.34)	.061
Pre-pregnancy BMI*	0.12	2.53 (1.03–6.22)	.043
Constant	−6.02	2.42e-03 (2.99e-5–0.20)	.007

*Note.* OR = odds ratio; BMI = body mass index.

* OR for continuous variables indicate the change in odds for an increase of one standard deviation.

**Figure 2:**
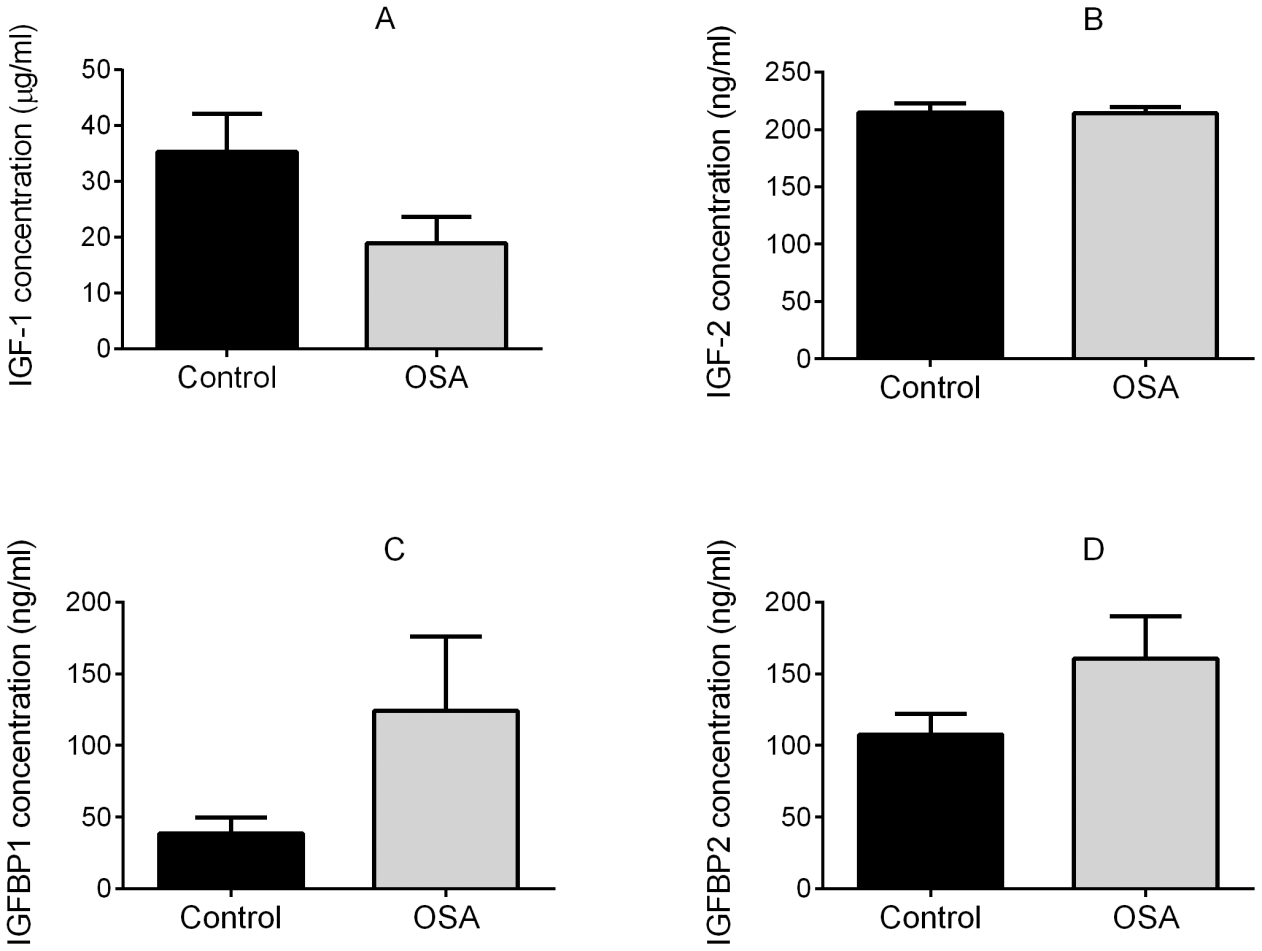
Fetal growth regulators (mean+/−SEM) in cord blood in controls (n = 21) and cases (OSA) (n = 10). **2A:** Insulin like growth factor 1 (IGF-1); **2B:** Insulin like growth factor 2 (IGF-2); **2C:** Insulin like growth factor binding protein 1 (IGFBP-1); **2D:** Insulin like growth factor binding protein 2 (IGFBP-2).

IGF-1 in the cord blood was lower among infants of cases compared to controls (mean 18.97 ug/mL (SEM 4.77) vs 35.31 ug/mL (SEM 6.88), p = 0.13) with a corresponding increase in IGFBP 1 (124.3 ng/mL (SEM 51.9) vs 38.8 ng/mL (SEM 10.8, p = 0.14) and IGFBP-2 (160.7 ng/mL (SEM 29.2) vs 107.5 ng/mL (SEM 14.5), p = 0.08). These results were in the direction expected, but were not significantly different (Figure 2A–D). There was no difference in IGF-2 between cases and controls; (mean 214 ng/mL (SEM 8.1) vs 214.9 ng/mL (SEM 8.1), p = 0.94).

Continuous overnight fetal heart rate monitoring was recorded for a mean of 6.7 (SD 1.5) hours among women undergoing polysomnography. Among women with OSA, the number of respiratory events recorded overnight ranged from 28 to 140. Fetal heart rate monitoring was captured for the majority of these events; a median of 80% of respiratory events were accompanied by successful fetal heart rate monitoring. Despite apnoeas/hypopnoeas of up to 30 seconds duration accompanied by maternal oxygen desaturation down to as low as 78%, these respiratory events were largely unaccompanied by acute fetal heart rate responses. The exception occurred in one fetus where two episodes of prolonged bradycardia were observed at 0213 hours and 0319 hours in a woman with proven OSA (RDI of 11.2). The first lasted for 2.5 minutes, and the second for approximately 4 minutes, and followed a period of frequent maternal hypopnoeas accompanied by significant oxygen desaturation (oxygen saturation nadir of 90%). A repeat cardiotocograph was normal the following morning and the treating clinician elected to resume routine weekly antenatal visits. She was delivered by elective repeat caesarean section at 38 weeks 5 days. At delivery, the baby's birth weight was 2640 g, and the customised birthweight centile was 1%.

## Discussion

We have demonstrated that OSA may be associated with impaired fetal growth in late pregnancy. In this study, we have found that even mild OSA may be associated with significant growth decrements. OSA may thus be an important, and previously unrecognised, contributor to stillbirth. The strength of our study is that we prospectively performed formal sleep studies to diagnose OSA. Previous studies examining the impact of OSA on fetal growth have mostly used a history of snoring alone, or screening questionnaires, as surrogates for OSA. They have yielded conflicting results [20], [21], [22], [23], possibly due to the previously reported poor specificity of screening questionnaires for detecting OSA in pregnancy, which have been confirmed again in this study [14]. Among studies that have used PSG to diagnose OSA, one large retrospective study reported an increased risk of low birth weight, small for gestational age (SGA) infants, preterm birth and low Apgar scores among women with OSA compared to controls [24], although these findings have not been consistently observed [25]. Collectively, these studies underscore that self reported symptoms using screening questionnaires alone are of limited value in predicting OSA and adverse outcomes in pregnancy. More robust studies, using polysomnography to confirm the diagnosis, and of appropriate size to evaluate the effects of confounding variables, are necessary to clarify the relationship between OSA and poor pregnancy outcome, and to identify which, if any, women would benefit from treatment.

The inconsistent results from previous studies may be partly due to the use of small for gestational age (birthweight less than the 10^th^ centile using population standards) as the sole measure of placental insufficiency. Assuming uteroplacental sufficiency when birthweight is above the 10^th^ centile has limitations in identifying the fetus that is failing to achieve its growth potential [26], [27], particularly among larger women. To more comprehensively evaluate the impact of OSA on fetal growth, we took three approaches. Firstly, we customised both the ultrasound estimate of fetal weight and birthweight. Fetal growth restriction is defined as a ‘failure to achieve growth potential’ [28], and customised growth charts better evaluate an individual fetus's growth potential by generating a fetal growth curve which is then modified for maternal characteristics, such as height, weight, parity and ethnicity. Customisation is particularly important in large women, where significant fetal growth restriction has occurred, but the fetus is not ‘small for gestational age’ at delivery (ie. less than the 10^th^ centile using population based birthweights). Fetal growth restriction defined by customised growth charts has been shown to be more strongly associated with adverse perinatal outcome, including stillbirth, compared with fetal growth restriction defined by population charts [29], [30], [31]. Secondly, we evaluated third trimester fetal growth trajectory since slowing of fetal growth across the third trimester may be an important indicator of uteroplacental insufficiency, even if the final birthweight is not below the 10^th^ centile. All fetuses born small due to placental insufficiency were once well grown, but whether the final birthweight is less than the 10^th^ centile at delivery will depend on variables such as the initial fetal size, the duration and severity of placental insufficiency, and the gestational age at delivery. Slowing of fetal growth can only be assessed with serial assessment of fetal size, and comparison of fetal and/or birthweight centile. In studies which have serially evaluated fetal growth, slowing of fetal growth trajectory has been shown to be associated with a higher risk of delivering a small for gestational age infant, with a corresponding increase in neonatal morbidity [32]. Further, in high risk pregnancies, those requiring operative delivery for fetal distress had a slower growth rate in the third trimester [33]. Among small for gestational age fetuses, those displaying a progressive fall in fetal growth are more likely to be severely growth restricted at birth and suffer adverse perinatal outcomes, including umbilical artery cord pH<7.1, admission to neonatal intensive care and emergency delivery for fetal distress [34], [35]. In low risk pregnancies, slowed third trimester growth has been shown to be associated with adaptive cerebral blood flow patterns associated with in utero hypoxia, and delivery outcomes consistent with those seen in infants with birthweight <10^th^ centile [36]. In clinical trials, slowing of fetal growth is recognised as an indicator of fetal growth restriction in late pregnancy [37]. Accordingly, in this study we wished to perform a comprehensive evaluation of fetal growth, including both the tracking of growth velocity in late pregnancy, as well as final birthweight centile.

Finally, we sought biochemical confirmation of growth impairment by measuring IGF-1, IGF-2 and their respective binding proteins in cord blood at delivery. The IGF axis is a critical growth regulation system, responsible for placental growth and differentiation, nutrient transfer and fetal growth [38]. Cord blood levels of IGF-1 in humans have been consistently demonstrated to be positively, and IGFBP-1 negatively, associated with birthweight [39]. IGFBP-2 levels in cord blood are also increased among fetuses with IUGR [40], [41]. Our biochemical data, while failing to achieve statistical significance, demonstrated trends in the expected direction corresponding with the observed fetal growth patterns among women with OSA, with a fall in IGF-1 and corresponding increase in IGFBP-1 and IGFBP-2.

In this study, we were able to successfully synchronise continuous electronic fetal heart rate monitoring with the sleep study, and record a satisfactory trace for the majority of the sleep period. Importantly, we have quantified the proportion of respiratory events that were accompanied by time synchronized fetal heart rate monitoring, confirming that the majority of these events in women with OSA occurred with the fetus under surveillance. Sahin et al first reported fetal heart rate decelerations in 3 out of 4 fetuses during maternal nocturnal oxygen desaturation in women diagnosed with OSA [19], but a more recent study involving 19 women with OSA, reported no association between fetal heart rate abnormalities and maternal respiratory events [42]. In this study, a mean of only 12.2 respiratory events per patient were accompanied by monitoring, compared to a mean of 43.3 events per patient successfully monitored in the current study. Reassuringly, we also found little evidence of acute fetal heart responses to maternal oxygen desaturation. The only fetus in our study that demonstrated an acute response to maternal oxygen desaturation was subsequently confirmed to have severe fetal growth restriction at birth. This raises the intriguing possibility that while most fetuses are well protected from transient maternal hypoxia, (perhaps due to a combination of fetal adaptive behaviours and the high affinity fetal haemoglobin-oxygen dissociation curve) episodes of maternal hypoxia may be poorly tolerated in fetuses with uteroplacental insufficiency. The potential impact of OSA on stillbirth may therefore be two-fold; chronic uteroplacental insufficiency resulting in impaired fetal growth, and acute fetal compromise provoked by frequent episodic maternal oxygen desaturation.

Our findings may be of considerable importance given the accumulating data linking obesity and stillbirth [43], and that obesity is a significant risk factor for OSA. The relationship between obesity and stillbirth has been attributed to co-morbidities such as hypertension and diabetes [44], or difficulty in diagnosing fetal growth restriction in obese women [45]. The findings of this study lead us to speculate that OSA may be an important mediator of the obesity-related stillbirth risk, through its effect on acute and chronic measures of fetal wellbeing. While the impact of OSA on fetal growth just failed to achieve statistical significance following adjustment for BMI, these outcomes suggest the association is still likely to be important. Indeed, it is possible that OSA may be a *mechanism* by which fetal growth is impaired in obese women, rather than obesity being a confounding variable; if so, adjusting for BMI may have underestimated the effect of OSA on fetal growth. If the trend observed in this study were confirmed in larger studies with the ability to adjust for all relevant confounding variables, it would be particularly exciting given that an effective intervention for OSA exists, in the form of Continuous Positive Airway Pressure (CPAP). In addition, CPAP has been already shown to be safe and well-tolerated in pregnant women in small studies [46].

In conclusion, we present evidence suggesting that the presence of objectively confirmed OSA in pregnancy may be associated with an adverse impact on fetal growth. If OSA is confirmed to be an important contributor to impaired fetal growth in larger studies, then OSA may also be a previously unrecognised cause of stillbirth. This raises the possibility of an in utero therapy for preventing and treating IUGR for which effective therapies are currently lacking, and- with it- a potential means of avoiding late pregnancy stillbirth.
